# Postoperative Outcomes of Bascom Cleft Lift Versus Excision With Secondary Wound Healing for Pilonidal Sinus Disease: A Multicenter Retrospective Analysis

**DOI:** 10.1097/DCR.0000000000003402

**Published:** 2024-08-08

**Authors:** Eleonora Anna Huurman, Jasper F. de Kort, Christel A.L. de Raaff, Maarten Staarink, Sten P. Willemsen, Robert M. Smeenk, Boudewijn R. Toorenvliet

**Affiliations:** 1 Department of Surgical Oncology and Gastrointestinal Surgery, Erasmus MC Cancer Institute, Rotterdam, the Netherlands; 2 Department of Surgery, Albert Schweitzer Hospital, Dordrecht, the Netherlands; 3 Department of Surgery, Ikazia Hospital, Rotterdam, the Netherlands; 4 Department of Surgery, Het Van Weel-Bethesda Hospital, Dirksland, the Netherlands; 5 Department of Epidemiology, Erasmus MC, Rotterdam, the Netherlands

**Keywords:** Bascom cleft lift, Complications, Excision with secondary wound healing, Pilonidal sinus disease, Successful wound healing

## Abstract

**BACKGROUND:**

Pilonidal sinus disease impacts a patient’s quality of life. In the Netherlands, it is often treated with excision and secondary wound healing, which is associated with high recurrence rates and poor wound healing. The Bascom cleft lift, an alternative technique, has shown favorable healing times and recurrence rates.

**OBJECTIVE:**

The present study compares successful wound healing, time to healing, complications, and recurrence rate between excision with secondary wound healing and Bascom cleft lift.

**DESIGN:**

This is a multicenter retrospective study.

**SETTINGS:**

Three institutions in the Rotterdam region of the Netherlands participated in the study.

**PATIENTS:**

Patients who underwent excision with secondary wound healing or Bascom cleft lift between July 2015 and August 2021 were included.

**MAIN OUTCOME MEASURES:**

Primary end points included the rate of successful wound healing and the time to achieve healing. Secondary end points included postoperative complications and the recurrence rate within 12 months after surgery.

**RESULTS:**

Of 272 patients, 128 underwent Bascom cleft lift and 144 patients underwent excision and secondary wound healing. Recurrent pilonidal sinus disease (47.7% vs 22.2%) and abscess history (53.1% vs 40.3%) were more common in the Bascom cleft lift group compared to excision with secondary wound healing. The median follow-up period at the outpatient clinic was 43 days. The wound healing rate was 84.4% after Bascom cleft lift versus 32.6% after excision and secondary wound healing (*p* < 0.001), with a median time to wound healing of 55 and 101 days, respectively (*p* < 0.001). Complications were 28.9% for Bascom cleft lift versus 13.2% for excision and secondary wound healing (*p* = 0.003). The rate of recurrent disease was 6.3% after Bascom cleft lift and 11.8% after excision and secondary wound healing (*p* = 0.113).

**LIMITATIONS:**

This study used a retrospective design, which makes it prone to selection bias and residual confounding. In addition, the short follow-up period adds to these limitations because a longer follow-up period may better identify true recurrence rates. The absence of collected patient satisfaction data, which is currently a common scientific issue, is also a deficiency.

**CONCLUSIONS:**

This retrospective study shows that Bascom cleft lift is superior to excision and secondary wound healing, given the higher percentage of patients with successful wound healing within a shorter time. See **Video Abstract**.

**RESULTADOS POSOPERATORIOS DE LA ELEVACIÓN DE LA HENDIDURA TIPO BASCOM VERSUS LA ESCISIÓN CON CICATRIZACIÓN DE LA HERIDA POR SEGUNDA INTENSIÓN PARA LA ENFERMEDAD DE SENO PILONIDAL: UN ANÁLISIS RETROSPECTIVO MULTICÉNTRICO:**

**ANTECEDENTES:**

La enfermedad de seno pilonidal afecta la calidad de vida del paciente. En los Países Bajos, a menudo se trata con escisión y cicatrización por segunda intensión, lo que se asocia con altas tasas de recurrencia y mala cicatrización de las heridas. La elevación de la hendidura de Bascom, una técnica alternativa, ha mostrado tiempos de curación y tasas de recurrencia favorables.

**OBJETIVO:**

El presente estudio tiene como objetivo comparar la cicatrización exitosa de la herida, el tiempo de cicatrización, las complicaciones y la tasa de recurrencia entre la escisión con cicatrización secundaria de la herida y la elevación de hendidura tipo Bascom.

**DISEÑO:**

Este es un estudio retrospectivo multicéntrico.

**AJUSTES:**

Tres instituciones en la región de Rotterdam de los Países Bajos participaron en el estudio.

**PACIENTES:**

Se incluyeron pacientes sometidos a escisión con cicatrización secundaria o elecación de la hendidura tipo Bascom entre julio de 2015 y agosto de 2021.

**PRINCIPALES MEDIDAS DE RESULTADO:**

Los resultados primarios incluyeron la tasa de curación exitosa de la herida y el tiempo para lograr la curación. Los resultados secundarios incluyeron complicaciones posoperatorias y tasa de recurrencia dentro de los doce meses posteriores a la cirugía.

**RESULTADOS:**

De 272 pacientes, 128 se sometieron a elevación de hendidura tipo Bascom y 144 pacientes a escisión y cicatrización secundaria. La ESP recurrente (47.7% frente a 22.2%) y los abscesos (53.1% frente a 40.3%) fueron más comunes en el grupo de elevación tipo Bascom en comparación con la escisión con cicatrización secundaria de la herida. La mediana del período de seguimiento en la consulta externa fue de 43 días. La cicatrización de la herida fue del 84.4 % después del lifting de Bascom frente al 32.6 % después de la escisión y la cicatrización secundaria (p < 0.001), con una mediana de tiempo hasta la cicatrización de la herida de 55 días y 101 días, respectivamente (p < 0.001). Las complicaciones fueron del 28.9% para el lifting tipo Bascom frente al 13.2% para la escisión y cicatrización secundaria (p = 0.003). La enfermedad recurrente fue del 6.3% después del lifting de hendidura tipo Bascom y del 11.8% después de la escisión y cicatrización secundaria (p = 0.113).

**LIMITACIONES:**

Diseño retrospectivo que lo hace propenso a sesgos de selección y confusión residual. Adicionalmente, el corto período de seguimiento del estudio aumenta aún más estas limitaciones, ya que un seguimiento más prolongado puede identificar mejor las verdaderas tasas de recurrencia. Por último, una deficiencia es la ausencia de datos recopilados sobre la satisfacción del paciente, lo que hoy en día es un problema científico común.

**CONCLUSIONES:**

Este estudio retrospectivo muestra que la elevación de hendidura tipo Bascom es superior a la escisión y cicatrización secundaria dado el mayor porcentaje de pacientes con curación exitosa de la herida en un tiempo más corto. *(Traducción—Dr. Jorge Silva Velazco*)

Pilonidal sinus disease (PSD) is a debilitating and difficult-to-treat condition. It is often seen in young patients and frequently results in decreased quality of life and absence from work or study.^1,2^ After surgical treatment, recurrences are seen in up to 20% of patients within 5 years.^3^ There is variation in surgical strategies for the treatment of PSD. Before the publication of the Dutch guideline on PSD,^4^ the most commonly used treatment in the Netherlands was wide local excision with primary or secondary closure.^4,5^ However, this treatment is associated with a high likelihood of wound breakdown (in case of primary closure), a long wound healing time, and a high recurrence rate.^3^ Several factors are associated with recurrent PSD, including the depth of the intergluteal cleft, the presence of moist conditions within the cleft, incomplete removal of affected tissue, tension on the wound edges after closure, and a midline suture. Therefore, new techniques have been developed, including the Bascom cleft lift (BCL). In 1990, Dr. John Bascom described this technique,^6^ in which the affected skin is excised with an elliptical incision and debridement of the sinus tracts is performed. The defect is then closed with a flap from the contralateral side, creating a tension-free closure that flattens the intergluteal cleft and shifts the suture away from the midline.^7,8^ The BCL has shown an overall success rate of 97%.^8–11^ However, there has been only 1 study directly comparing the BCL to wide excision with secondary wound healing (ESW). This study was conducted among adolescents with a limited number of patients (n = 70).^12^ The present study aims to assess and compare the incidence of successful wound healing, time to wound healing, complications, and recurrence rate between ESW and the BCL.

## MATERIALS AND METHODS

This is a multicenter retrospective study of patients who underwent a BCL or ESW at 3 institutions in the Rotterdam region of the Netherlands. These 3 hospitals have adopted the BCL as one of the surgical treatment modalities for PSD primarily as an alternative to ESW. Study approval was obtained from the Medical Research Ethics Committee Utrecht. Informed consent was not obtained because requesting permission would have required a significant amount of time and effort because of the large numbers of patients who underwent operations several years ago. This is in accordance with the exception rules. This multicenter retrospective study was conducted according to the Strengthening the Reporting of Observational Studies in Epidemiology guidelines.

### Patients

All patients aged 16 years or older with symptomatic PSD who underwent a BCL or ESW between July 2015 and August 2021 were selected for inclusion. If patients underwent 2 operations during the study period, the first operation was included for analysis. No patients were excluded. Patient-specific characteristics, including age, sex, obesity (BMI ≥30), prior PSD surgical procedures, number of midline pits, lateral sinus openings, and comorbidities (diabetes, use of immunosuppressive) and smoking status were collected from patient medical records. Operative reports were studied to ensure similar operative techniques were used.

### Evolution of Surgical Techniques

Before 2015, the standard approach in all three institutions for the surgical treatment of PSD involved wide excision of all affected tissue, usually followed by wound healing through secondary intention. In 2015, a surgeon introduced the BCL at one of the institutions (hospital 1) as an alternative treatment option for PSD. Two other institutions adopted this technique in 2017 (hospital 2) and 2019 (hospital 3). The surgeon in hospital two began using BCL after hearing a presentation and using online video instructions. The surgeon in hospital three had on-site proctoring by the surgeon from hospital one.

The BCL procedure involved excising the affected skin with an elliptical incision and performing debridement of the sinus tracts. A skin flap was raised on the side opposite the excision, and the incision was modified as needed to ensure proper closure away from the midline. The defect was then closed with a flap from the contralateral side of the affected area. The flap was sutured in place, and the skin was closed with absorbable sutures. The space between the deep closure and the flap was drained with a 12-French channel drain in hospitals 1 and 3, whereas 2 vessel loops were used in hospital 2, brought out on the upper buttock on the flap side. The drain was removed sometime after postoperative day 3, as long as the output was <10 mL per 24 hours. The surgeries were conducted as day procedures. Routine antibiotics were administered to the BCL patients in hospital 1.

### Outcomes

Outcomes were the successful wound healing rate (complete wound/skin closure with no residual drainage or symptoms at the last outpatient visit), the time to achieve wound healing (days), the incidence of postoperative complications and disease recurrences (reappearance of the disease and/or symptoms within 12 months after surgery), as well as abscesses and reoperations. These complications included postoperative bleeding (bleeding that occurs either immediately or delayed after surgery), hypergranulation (excessive formation of granulation tissue), seroma (recurrence of fluid collection after drain removal after BCL or occurrence of a fluid collection after partial closure after ESW), wound abscess (localized collection of pus), wound dehiscence (disruption of suture line leading to distraction of opposing wound edges) and wound infection (an open wound with pus). Disease recurrences were evaluated on the basis of medical records up to 1 year postsurgery.

### Statistical Analyses

Statistical analysis was performed using SPSS version 24.0 or higher (SPSS Inc, Chicago, IL). Continuous variables were evaluated for normality using visual inspection. For continuous variables, normally distributed data were reported as means ± SDs, and medians with interquartile ranges were used in skewed data. Categorical data were reported as frequencies and percentages. Associations with continuous variables and types of treatment were analyzed using the ANOVA test in the case of normally distributed data and the Kruskal-Wallis test in the case of nonnormally distributed data. Associations with categorical variables and type of treatment were analyzed with the χ^2^ test. The time until wound healing was visualized using the Kaplan-Meier method and analyzed using the log-rank test. To account for possible confounding by abscess history, primary/recurrent disease, and type of treatment, we used a Cox proportional hazards model to estimate the time until wound healing. A univariable regression analysis was conducted to compare outcomes (complications, wound dehiscence, recurrences, reoperations) between hospitals. For significant differences, additional analyses were performed using multivariable analysis. Clinical variables believed to be potentially influential were included depending on the outcome.

## RESULTS

### Patients

Two hundred seventy-two patients were included (Table 1). Among them, 217 (79.8%) were men and 55 (20.2%) were women. The mean (SD) age was 32 (10.8) years, and the mean (SD) BMI was 27 (5.0). A total of 128 BCL and 144 ESW procedures were performed. The majority of patients (65.8%) had primary PSD, which was more common in the ESW group.

**TABLE 1.** Patient characteristics (N = 272)

*Characteristics*	*BCL (N = 128*)	*ESW (N = 144*)	*p*
Male, n (%)	106 (82.8)	111 (77.1)	0.24
Age, mean (SD)	30 (9.6)	34 (11.5)	0.65
Primary PSD, n (%)	67 (52.3)	112 (77.8)	**<0.001**
Abscess history, n (%)	68 (53.1)	58 (40.3)	**0.03**
BMI, mean (SD)	27 (5.2)	27 (4.8)	0.56
Smoking, n (%)	55 (42)	46 (31.5)	0.06
Diabetes, n (%)	0 (0)	4 (2.8)	0.057
Immunosuppressive use, n (%)	0 (0)	2 (1.4)	0.18
Midline pits, mean (SD)	2.8 (1.8)	NA	NA
Lateral sinus openings, n (%)	66 (68.7)	NA	NA

Data are mean and SD and frequencies and percentages. Bold values indicate statistically significant differences (*p* < 0.05) compared to the control or reference group.

BCL = Bascom cleft lift; ESW = excision with secondary wound healing; NA = not applicable; PSD = pilonidal sinus disease.

### Outcomes

#### Wound healing

Out of the 128 BCL operations performed, 108 (84.4%) resulted in successful wound healing during the last outpatient visit, with a median follow-up period of 45 days. However, 60 patients in the BCL group experienced wound dehiscence, of whom 40 had closed skin at the last outpatient visit (Table 2). The median time to wound closure after wound dehiscence was 80 days for these patients. Among all cases of wound dehiscence, 80% were classified as small (≤2 cm), located caudally in the wound. All of these cases were managed by flushing daily.

**TABLE 2.** Characteristics of wound dehiscence in the BCL group (n = 60)

*Characteristic*	*n (%*)
Localization	
Cranial	2 (3.3)
Caudal	39 (65)
Cranial and caudal	3 (5.0)
Caudal and middle	1 (1.7)
Middle	5 (8.3)
Unknown	10 (16.6)
Wound dehiscence size (length), cm	
<2	48 (80)
2–5	6 (10)
>5	6 (10)
Skin closed after wound dehiscence at last outpatient visit	40 (66.7)

BCL = Bascom cleft lift.

**FIGURE 1.**
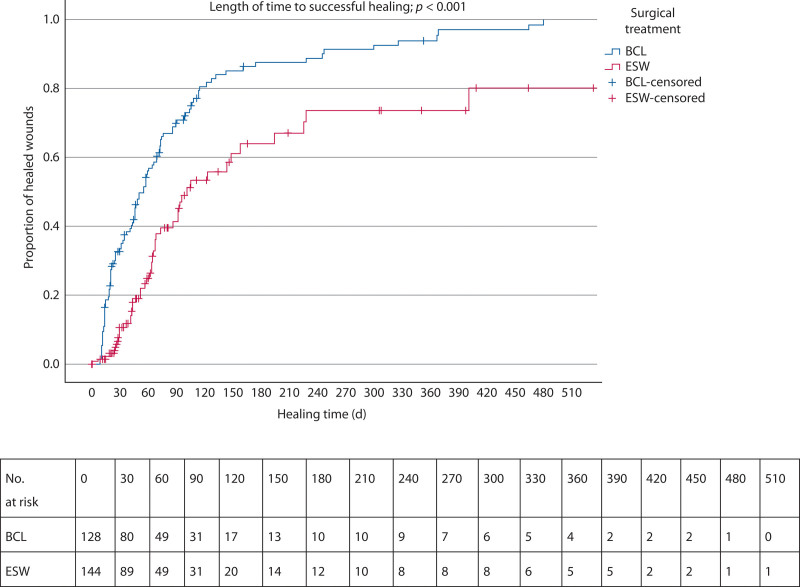
Kaplan-Meier curve demonstrating the time to successful healing after BCL and ESW and number at risk tables. BCL = Bascom cleft lift; ESW = excision with secondary wound healing.

In the ESW group, 47 of 144 patients (32.6%) had successful wound healing, with a median follow-up period of 41 days, which was statistically lower compared to the BCL group (*p* < 0.001, OR 0.358 [95% CI, 0.249–0.514]; Fig. 1). Five patients were lost to follow-up in this group. The median time to successful wound healing was 55 days (95% CI, 45–65) for the BCL, compared to 101 days (95% CI, 69–133) for ESW (*p* < 0.001).

#### Postoperative complications

In the BCL group, 37 patients experienced complications (28.9%), whereas in the ESW group, 19 patients experienced complications (13.32%, *p* = 0.003). Postoperative complications adjusted for confounding factors (abscess history, primary/recurrent disease, type of treatment, and type of hospital) are presented in Table 3. This includes details of the treatments provided.

**TABLE 3.** Postoperative complications

	*BCL (N = 128), n (%*)	*ESW (N = 144), n (%*)	*p*	*Exp (B) (95% CI*)
Complication, n (%)	37 (28.9)	19 (13.2)	**0.003**	0.424 (0.222–0.808)
Bleeding^a^	2 (1.6)	5 (3.5)		
Hypergranulation^b^	1 (0.8)	1 (0.7)		
Seroma^c^	9 (7.0)	1 (0.7)		
Wound abscess^d^	0 (0)	1 (0.7)		
Wound dehiscence (>2 cm)^e^	12 (9.4)	0 (0)		
Wound infection^f^	13 (10.2)	11 (7.6)		

Data are frequencies and percentages. Bold values indicate statistically significant differences (*p* < 0.05) compared to the control or reference group.

BCL = Bascom cleft lift; ESW = excision with secondary wound healing.

a BCL: 1 pressure application, 1 reexploration in operating room; ESW: 4 sutures, 1 pressure application.

b BCL/ESW: silver nitrate.

c BCL: 4 conservative, 3 needle aspirations, 1 antibiotics, 1 small incision; ESW: conservative.

d ESW: spontaneously drained.

e BCL: 10 flushings (3 + terracotryl), 1 debridement and suturing, 1 split-skin graft.

f BCL: 13 antibiotics; ESW: 8 antibiotics, 3 flushings.

#### Hospital variation

Hospitals 2 and 3 had a statistically significant higher rate of wound dehiscence compared to hospital 1 (Table 4; *p* < 0.001), a finding that remained after multivariable regression analysis, including BMI, abscess history, primary/recurrent disease, and the type of hospital. BMI was found to be a statistically significant predictor of wound dehiscence.

**TABLE 4.** Postoperative outcomes between hospitals (N = 131)

*Outcomes, n (%*)	*Hospital 1*	*Hospital 2*	*Hospital 3*	*p*
Complications	23/75 (30.7)	7/34 (20.6)	7/19 (36.8)	0.40
Wound dehiscence	25/75 (33.3)	18/34 (52.9)	17/19 (89.5)	**<0.001**
Recurrences	4/75 (5.3)	1/34 (2.9)	3/19 (15.8)	0.16

Bold values indicate statistically significant differences (*p* < 0.05) compared to the control or reference group.

#### Recurrence

**FIGURE 2.**
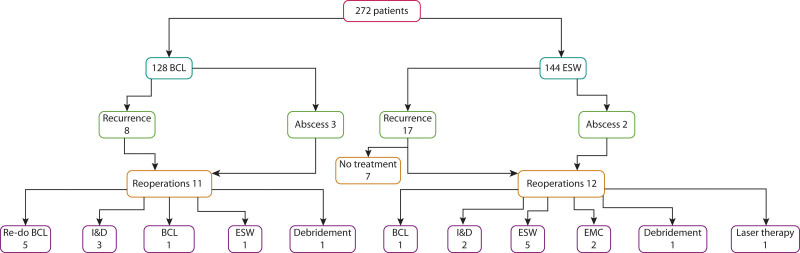
Recurrences. BCL = Bascom cleft lift; EMC = excision with midline closure; ESW = excision with secondary wound healing; I&D = incision and drainage

**FIGURE 3.**
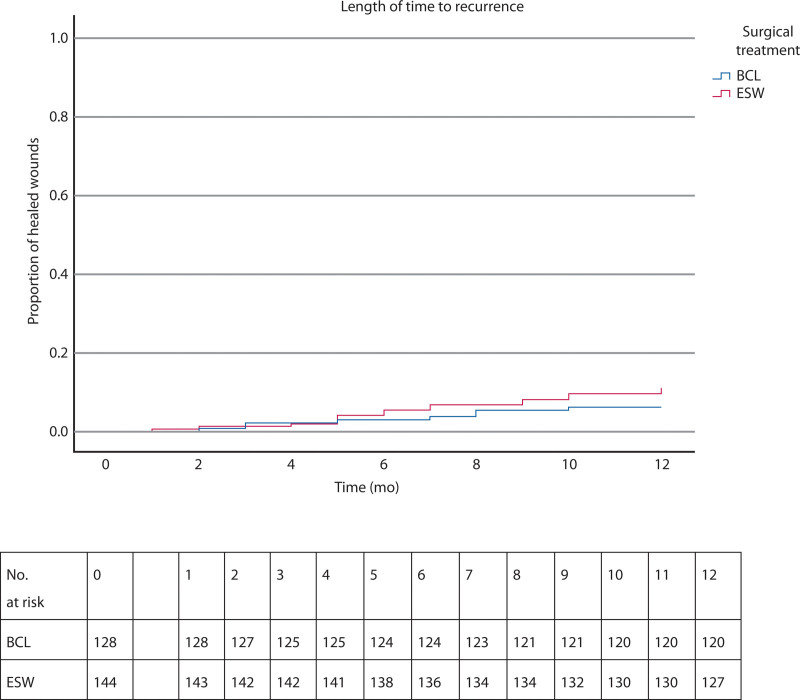
Kaplan-Meier curve demonstrating the time to recurrence after BCL and ESW and number at risk tables. BCL = Bascom cleft lift; ESW = excision with secondary wound healing.

In the BCL group, 8 of 128 patients had a recurrence (6.3%) compared to 17 of 144 patients (11.8%) in the ESW group within the first year after surgery (*p* = 0.113; Figs. 2 and 3). When calculating recurrences only after successful wound healing, the percentages were as follows: 2 of 108 patients (1.9%) for BCL and 5 of 47 patients (10.6%) for ESW, with a significant difference observed (*p* = 0.015).

#### Overall outcomes

In the ESW group, 97 patients failed to achieve successful wound healing, with an additional 5 experiencing recurrence, resulting in an unfavorable outcome for 102 of 144 (70.8%) patients. In contrast, the BCL group had 20 patients who failed to achieve successful wound healing, with 2 experiencing recurrence, leading to an unfavorable outcome in 22 of 128 (17.2%) patients (*p* ≤ 0.001).

## DISCUSSION

The results of this multicenter retrospective study suggest that the BCL is superior to ESW for both primary and recurrent diseases. The BCL resulted in a significantly higher percentage of wound healing compared to the ESW group (84.4% versus 32.6%). In addition, the median time to successful wound healing in the BCL group was only half that of the ESW group (55 versus 101 days). It should be noted that there was still a considerable amount of time after surgery until complete wound healing/skin closure was achieved. Furthermore, there are fewer recurrences within 12 months after the BCL. An explanation for this could be that the BCL flattens the intergluteal cleft, which is one of the risk factors for recurrent PSD.^13^ Within the BCL group, there were more patients with recurrent PSD and patients who had previously undergone abscess incision and drainage. An explanation may be that BCL is most often used for complex or recurrent PSD. This may also account for the more frequently observed complications, including wound dehiscence, after BCL. Primary skin closure has a higher risk of complications/infections. In addition, it is worth noting that most of the wound dehiscences were small and had closed by the last outpatient visit.

If we compare the outcomes between the hospitals, hospital 3 experienced a significantly higher rate of wound dehiscence after the BCL. Reasons for this could still include differences in patient populations and possible modification of the operative technique. There may be other risk factors for wound dehiscence that we did not include in the multivariable analysis. The learning curve may also play a role, although no definitive conclusions could be drawn about the learning curve of the BCL based on the results in this study because of the relatively small number of patients. Hospital 3 performed the lowest number of BCL procedures and had the least experience with the operative technique.

Despite the higher complication and wound dehiscence rate after the BCL, patients might choose to undergo this procedure because of its potential to achieve a higher percentage of wound healing in a shorter time and with fewer recurrences. Immerman^9^ stated that BCL is a remedy for all types of PSD given the overall success rate of 96.6% and the high level of patient satisfaction reported in his experience. The recent Dutch guideline (2022) aligns with this perspective and no longer recommends ESW.^4^ This present study shows a recurrence rate of 6.3%. A single surgeon experience with BCL by Ojo et al^10^ described similar recurrence rates of 5.3% after the BCL. This is also consistent with the findings of Stauffer et al, who observed that the BCL (as well as Karydakis) had the lowest recurrence rate of all procedures for PSD during various follow-up periods.^14^ The superiority of the BCL with respect to ESW was also evident in two other studies that compared the BCL to ESW and excision with midline closure, with a significantly higher percentage of wound healing and a reduced wound healing time in the BCL group.^12,15^ Taking all of this into consideration, it is advisable for surgeons to seriously contemplate the BCL as a treatment option for PSD. The BCL still outperformed ESW in our study, but the results of our BCL procedures were less favorable than those reported in the literature.

### Limitations

This study has its limitations. Its retrospective design makes it prone to selection bias and residual confounding. In addition, the short follow-up period of the study further adds to these limitations because longer follow-ups may better identify true recurrence rates. Finally, a deficiency is the absence of collected patient satisfaction data, which is currently a common scientific issue.

## CONCLUSIONS

ESW should be discontinued because of the poor results of this procedure in this study. Future prospective studies are necessary to explore the long-term outcomes and overall satisfaction after the BCL for PSD. Given the promising results compared to ESW, it seems sensible to allocate additional time and resources to optimize and standardize the BCL technique and evaluate its performance in prospective studies after implementation in centers that treat patients with PSD.
